# Attention deficit hyperactivity disorder diagnoses and prescriptions in UK primary care, 2000–2018: population-based cohort study

**DOI:** 10.1192/bjo.2024.781

**Published:** 2024-09-19

**Authors:** Alexandra Philipsen

**Affiliations:** Department of Psychiatry and Psychotherapy, University Hospital Bonn, Bonn, Germany

**Keywords:** Attention-deficit hyperactivity disorder, administrative prevalence, attention-deficit hyperactivity disorder medication, primary care

## Abstract

Attention-deficit hyperactivity disorder (ADHD), a common developmental disorder, affects 5–7% of children and 2.5% of adults globally. Recent increases in ADHD medication prescriptions have sparked the debate on overdiagnosis and overtreatment. McKechnie et al. examine UK ADHD prevalence and medication trends over 18 years, with implications for mental health services.

Attention-deficit hyperactivity disorder (ADHD) has a global prevalence of 5–7% in children and adolescents^1–3^ and often persists into adulthood.^4^ Characterised by symptoms of age-inappropriate in attention, hyperactivity and impulsivity, ADHD places a significant burden on patients as it is often associated with psychosocial, educational and neuropsychological impairment.^5,6^

Individuals with ADHD are at higher risk for several negative outcomes, including affective disorders,^7^ substance misuse,^8^ traffic accidents,^9^ injuries and emergency department visits^,10^ criminal behaviour^11^ and suicidality.^12^

The aetiology of ADHD is multifactorial, complex and heterogeneous. ADHD is thought to be an accumulation and correlation of mainly genetic but also environmental risk factors (e.g. low birth weight^13^ as well as the interaction of gene × environment).^14,15^

Children with ADHD are disproportionately affected by socioeconomic disadvantage^16^ and ADHD is associated with multiple adverse childhood experiences.^17^

Awareness of the condition and appropriate treatment are important for people with ADHD and their families.

## Recent global prevalence data

Recent estimates based on 53 studies suggest an even higher global prevalence rate of 7.6% for children and 5.6% for adolescents, with boys twice as likely to have ADHD.^18^ Meta-regression analysis has shown that the variability in ADHD prevalence is caused by methodological differences between studies, diagnostic criteria, sources of information and the requirement of functional impairment for diagnosis, which varies between regions and countries.^1,19–21^

Longitudinal studies of children with ADHD show a general decline in symptoms over time. However, the majority of patients with childhood-onset ADHD continue to have symptoms into adulthood.^4^ In particular, studies of health insurance data have impressively demonstrated a critical treatment gap in the transition period from adolescence to young adulthood.^22^

Studies such as McKechnie et al^23^ are warranted given the population-based prevalence of ADHD, the impact of ADHD on individuals and their families and the ongoing debate about under- or overdiagnosis and treatment^24,25^. This is particularly important, as the debate may cause additional distress to individuals and their families and increase stigma and prejudice.

## McKechnie study

The study by McKechnie et al in 2023^23^ addressed the lack of administrative incidence and prevalence rates of ADHD diagnosis and prescription in adults and children in the UK. The authors estimated incidence and prevalence rates using electronic health records of 7 655 931 individuals (IQVIA medical research data) routinely collected from UK primary care.^23^

It is important to note that data on non-pharmacological treatments, such as psychoeducation or behavioural interventions that are part of a holistic approach to ADHD, were not included in this analysis.

ADHD diagnosis and prescription rates were calculated between 1 January 2000 and 31 December 2018 for patients aged 3–99 years, including age, gender, social deprivation and calendar year, using descriptive statistics, regression analysis and time series analysis. To be included in the analyses, individuals had to be permanently registered with a participating practice within the time period analysed, and only individuals with data for at least 1 full calendar year were included.

Social deprivation was calculated using the Townsend deprivation index. The index includes a measure of the multiple of deprivation in a given geographical area. The measures included in this study were unemployment, car ownership, home ownership and household overcrowding. Scores were defined for areas of around 150 households and grouped into quintiles.

Of the 7 655 931 individuals included in the analyses, 0.5% had a diagnosis of ADHD and 0.2% received at least two prescriptions for ADHD medication from primary care within 1 year. Notably, of the 35 877 individuals with a diagnosis of ADHD, 57% did not receive a prescription for ADHD medication from primary care.

## New diagnoses

New diagnosis rates were highest among children aged 6–9 and young adults aged 18–29. In addition, new diagnosis rates were highest in the most deprived areas for both children and adults. Rates of first prescription of ADHD medication followed a similar pattern.

According to data from the USA and Germany,^26^ overall, the rate of new ADHD diagnoses increased between 2000 and 2018 for both genders. In boys, it doubled, while in men it increased almost 20-fold. In girls, the rate of new ADHD diagnoses increased fourfold, while in women it increased 15-fold.

## Prevalence of ADHD diagnoses and prescriptions

In boys aged 3–17, the overall proportion of ADHD diagnoses was 1.8%, compared with 0.4% in girls. In adult males aged 18–99, the overall rate was 0.3%, while in females it was 0.07%.

In children, the proportion of ADHD medication use increased between 2000 and 2018 and was 0.9% in boys (a fourfold increase) and 0.2% in girls (a ninefold increase). In the adult population, this proportion of ADHD medication use increased to 0.04% in males (30-fold increase) and 0.01% in females (15-fold increase). ADHD medication use peaked in the 10–15 age group and declined in older groups (despite higher ADHD prevalence rates).

Contrary to the trend of increasing diagnoses and treatments in all age groups, only children under 6 years of age showed a decrease in new diagnoses and treatments.

## Implications for health services

The available data now show neither overdiagnosis nor overtreatment with ADHD medication^27^ in primary care in the UK, compared with the expected population-based prevalence of ADHD. Although there has been an increase in diagnoses, reflecting increased awareness, the administrative prevalence is still very low, indicating that awareness is still insufficient.

The gender ratio of new diagnoses of ADHD in adult men and women found in the study by McKechnie et al^23^ (1:5 to 1 from age 30, 1:1 from age 40) reflects the balanced gender ratio of adult ADHD known from epidemiological studies.

National Institute for Health and Care Excellence (NICE) guidelines and education activities, such as the UK Adult ADHD Network (UKAAN), are likely to have had an impact on these changes.

However, ADHD still seems to be underdiagnosed and undertreated, especially in adulthood, in both genders, but particularly in women compared to men.

As with other mental disorders, the symptoms of ADHD overlap with those of other diagnoses, such as borderline personality disorder.^28,29^ Diagnostic attention should be paid to possible (additional) ADHD, especially in women.

In addition, this study highlights again the transition gap, that is, the complications in accessing transitional care for adolescents between child and adult services.^22,30–32^

The study emphasises the importance of strengthening educational and service planning and allocating resources. Equitable access to ADHD diagnosis and treatment is of paramount importance. This applies to children, particularly those from disadvantaged backgrounds,^33^ as well as to adolescents in transition and adults, where it is known that symptoms often persist into adulthood.

In conclusion, the study by McKechnie et al^23^ is an important step in providing further valuable guidance for primary care in the UK and beyond.

## Data Availability

Data availability is not applicable to this article as no new data were created or analysed in this study.
